# Cerebrovascular Complications After Heart Transplantation

**DOI:** 10.2174/157340310791658811

**Published:** 2010-08

**Authors:** Aída Alejaldre, Raquel Delgado-Mederos, Miguel Ángel Santos, Joan Martí-Fàbregas

**Affiliations:** Stroke Unit, Neurology Department, Hospital de la Santa Creu i Sant Pau, Spain

## Abstract

Neurological complications in orthotopic heart transplantation represent a major cause of morbidity and mortality despite successful transplantation. The most frequent perioperative neurological complications are delirium or encephalopathy. In this period cerebrovascular complication ranges between 5-11%. After the perioperative period, the 5-year stroke risk after cardiac transplantation is 4.1%. In a retrospective study conducted with 314 patients who underwent cardiac transplantation, it was found that 20% of cerebrovascular complications occurred within the first two weeks after transplantation, while 80% occurred in the late postoperative phase. Of these, ischemic stroke is the most common subtype.

In the perioperative periode, hemodynamic instability, cardiac arrest, extracorporeal circulation over 2 hours, prior history of stroke, and carotid stenosis greater than 50% have been reported to be risk factors for the occurrence of cerebrovascular complications. Perioperative cerebrovascular complications are associated with higher mortality and poor functional outcome at one year follow-up.

After the perioperative period, the only factor that has been significantly associated with an increased risk of cerebrovascular complications is a history of prior stroke, either ischemic or hemorrhagic. Other associated factors include unknown atrial fibrillation, septic emboli from endocarditis, cardiac catheterization and perioperative hemodynamic shock. According to the TOAST etiologic classification, the most prevalent etiologic subtype of ischemic stroke is undetermined cause.

## INTRODUCTION

In recent decades, organ transplantation is a therapeutic alternative with excellent results in quality of life and survival. Cardiac transplantation is a currently accepted treatment for many patients with terminal heart disease. Since the first heart transplant performed by Bernard in 1967, improvement in surgical techniques as well as immunosuppression has led to increasing survival rates up to 80% in the first year, and 70% at 5 years [1]. For this reason, heart transplantation is an elective treatment for patients with advanced irreversible cardiopathies with no other feasible medical or surgical options. 

However, cardiac transplantation is not without complications. The most common causes of mortality in cardiac transplantation are infections, graft vascular disease, sudden death, acute graft failure, tumours and acute rejection [1].

Neurological complications in orthotopic heart transplanttation represent a major cause of morbidity and mortality despite successful transplantation. The incidence and characteristics of neurological complications vary according to studies, depending on whether they are diagnosed at autopsy or clinically in survivors. According to some reports, the incidence of neurological complications in patients with cardiac transplantation varies from 23% in the perioperative period [2] to 13% in the first month [3], and 18% in the first ten years after transplantation [4].

Table **1** shows the frequency of neurological complications in the perioperative period, from day 1 to 60 days after transplantation. Neurological complications in this period are influenced by the underlying disease, the surgical procedure and surgical or medical complications during postoperative course. According to published series, the most frequent perioperative neurological complications are delirium or encephalopathy (9% of transplant patients in some series) [2]. Cerebrovascular complication during this period ranges between 5-11% [2, 4]. Of these, ischemic stroke is the most common subtype.

Table **2** shows the frequency of neurological complications after the perioperative period which may occur in up to 72% of recipients in long term follow-up [2]. Due to neurologic complications, mortality is 8% of patients during the first year and 12% of survivors suffer a moderate functional dependence. Despite this, 80% of patients who suffer neurological complications after cardiac transplantation have a good functional outcome in the first year [2].

### Cerebrovascular Complications

A retrospective review of 314 patients who underwent cardiac transplantation showed that 20% of cerebrovascular complications occurred within the first two weeks after transplantation, while 80% occurred after this period [5].

The most common cerebrovascular complication is ischemic stroke. Other cerebrovascular complications reported include transient ischemic attack (TIA), hemorrhagic stroke, anoxic encephalopathy, hyperperfusion syndrome or cognitive impairment of vascular etiology (Table **3**).

It has been suggested that the etiology of heart disease leading to transplantation could be related to the risk of post transplantation cerebrovascular complications. In some studies, dilated cardiomyopathy, ischemic cardiomyopathy, and valvular heart disease were found to be the most frequent etiologies implicated [3, 4]. However, other studies did not found this association [5]. Unexpectedly, established risk factors of stroke such as history of atrial fibrillation or presence of intracardiac thrombus were not related to the incidence of post transplantation cerebrovascular complications [6].

The only vascular risk factor associated with an increased risk of cerebrovascular complications after heart transplantation is the history of previous ischemic or hemorrhagic stroke [5]. In the following sections we review these cerebrovascular complications in two different periods: the perioperative period and the long-term follow-up period.

### Intraoperative and Perioperative Period

Cerebrovascular complications occurring within the intraoperative and postoperative period are associated with high mortality in the first year after transplantation. Different factors may play an important role in the development of cerebrovascular complications in this period and therefore the etiology of cerebrovascular complications is often multifactorial (asystole, cardiac trauma, gaseous embolism, fat embolism, aortic or cardiac embolism, global ischemia, coagulation disorders, infections) [5, 7].

Hemodynamic instability, cardiac arrest [6], extra-corporeal circulation over 2 hours [8], prior history of stroke, and carotid stenosis greater than 50% [9] have been reported to be decisive for the appearance of cerebrovascular complications [6]. Clamping and manipulation of the ascending aorta and the type of surgical technique (standard versus bicava) have been also associated with higher risk of cerebrovascular events, but without statistical significance. 

Within the intraoperative period, global cerebral hypoxia may occur due to intraoperative complications such as right heart failure or ventricular laceration. Both ischemic and hemorrhagic stroke are associated with aortic embolism due to handling, incomplete reversal of antiplatelet/anticoagulant treatment, and elevated pressure in the cardiopulmonary bypass [7].

Ventricular assist devices are another important risk factor for cerebrovascular events. In a prospective study, up to 66% of cerebrovascular complications occurred within 4 months after implantation of the device. The stroke risk is greater with longer ventricular assist device support period. On the other hand, it has been reported that 42% of strokes occur in patients with concomitant infections presumably due to platelet activation which predisposes to stroke. Selection of ventricular assistance, infection prevention and strict control of anticoagulation in these cases can prevent these complications [10].

In this period, patients who undergo cardiac catheterization must be taken into account, as these represent an estimated rate 8.3% of strokes events [5, 6]. 

Moreover, disseminated intravascular coagulopathy (DIC) may occur during this period. DIC has been diagnosed in patients with sepsis caused by various microorganisms after heart transplantation. These patients are at an added risk of cerebral parenchymal haemorrhage and subdural hematomas [7]

Another cerebrovascular complication is the hyperperfusion syndrome. In patients with chronic cardiac pump failure, a reduction of cerebral blood flow has been described in transcranial Doppler and SPECT studies. These alterations in brain perfusion can rapidly normalize in patients following cardiac transplantation. In a study by Massaro *et al.* the mean blood flow velocity of the middle cerebral artery and the pulsatility index was measured by transcranial Doppler. In the Preoperative period, patients had a mean flow velocity of the middle cerebral artery of 45.1 cm/sec and a Pulsatility Index (PI) of 0.98. In successfully transplanted patients, the mean velocity increased by 53.3% (p<0.0001) and mean PI increased 2.6% (NS). Furthermore, dicrotic waves, observed in most patients before the transplant as a possible marker of refractory heart failure, normalized afterwards. Some of the patients that showed a rapid increase of the flow velocity presented symptoms suggestive of hyperperfusion syndrome consisting of severe headache, nausea, vomiting and decreased level of consciousness. Thus, the rapid improvement of cardiac function after transplantation may be the cause of the hyperperfusion syndrome [11].

### Follow-Up of the Transplanted Patient

Ischemic stroke is the most prevalent cerebrovascular complication during the follow-up after heart transplantation. After the perioperative period, the only factor that has been significantly associated with an increased risk of cerebrovascular complications is a history of prior stroke, either ischemic or hemorrhagic [5]. No increased risk of cerebrovascular complications was found in patients with atrial fibrillation, anemia, hypotension or hypertension and cardiac pump failure. 

In the report by Belvis *et al*. [5], it was found that the most frequent clinical syndrome, according to the classification of the Oxfordshire Community Stroke Project, was the partial anterior circulation syndrome (Table **4**).

Stroke of undetermined cause, according to the TOAST etiologic classification, is the most prevalent etiologic subtype of ischemic stroke (Table **5**). In Belvis’s *et al.* report, unknown atrial fibrillation was the cause of all cardioembolic strokes, while septic emboli in bacterial endocarditis was identified as a causal factor in other reports [5, 7].

The unusual etiology ischemic strokes were caused by cardiac catheterization and perioperative hemodynamic shock [5]. 

TIA is the second most common cerebrovascular complication. TIA after heart transplantation has not been associated with any cause or predisposing factor.

On the other hand, hemorrhagic stroke is an unusual cause of cerebrovascular disease after heart transplantation. In some studies hemorrhagic stroke has not been observed as a cerebrovascular complication outside perioperatory period. However, in other studies, it has been described as the cause of up to 12 % of cerebrovascular complications. A history of previous hemorrhagic stroke has been associated with an increased the risk of cerebral bleeding complication [5]. Furthermore, patients who present a TIA have a higher risk of hemorrhagic stroke in the follow-up [5].

In patients with a history of pre-transplant ischemic or hemorrhagic stroke, the 5-year stroke risk after cardiac transplantation, is 4.1%, which is significantly higher than in non-transplanted patients [5].

Immunosuppressive treatment is an additional factor that can be responsible of neurological complications after cardiac transplantation. It is difficult to evaluate the relationship between risk of ischemic stroke and immunosuppressive drugs, because there are numerous drug combinations in the immunosuppressive regimen.

Sirolimus is a potent immunosuppressive drug that often is part of the immunosuppressive regimen in heart transplant patients. Sirolimus as a part of combined immunosuppressive regimen has been identified as a potential cause of thrombotic microangiopathy. Frequent neurological complications associated with Sirolimus include polyneuropathy, tremor and ischemic stroke, bleeding and TIA.

However in a study by Diederik *et al.* [12], patients who suffered cerebrovascular complications after cardiac transplantation were not under treatment with Sirolimus. Based on this observation, the authors speculated that Sirolimusbased immunosuppressive therapy may prevent cerebrovascular complications. However more studies are needed to confirm this hypothesis. 

## CONCLUSION

Neurologic complications after cardiac transplantation are a major cause of morbidity. Among neurological complications, cerebrovascular disease occurs more frequently after the perioperative period, and the most common type is ischemic stroke. There is no clear relationship between the specific heart disease that lead to transplantation and cerebrovascular complications. However, it is well known that a previous history of stroke is an important risk factor to suffer a recurrent stroke after transplantation. The most common etiologic stroke subtype is undetermined, as in stroke that affects non-transplanted patients. 

When cerebrovascular complications occur in the perioperative period, a high mortality within the first year has been described. A proper selection of the patient and the assessment of the most appropriate surgical technique are very important. It is also essential to have a good neurological evaluation after anaesthesia because some focal cerebrovascular diseases are undiagnosed, and should be treated as soon as possible.

In conclusion we must keep in mind the importance of cerebrovascular complications in transplanted patients. In addition to the mortality associated with these events, there is also the risk of poor functional outcome in 12% of patients at the 1-year follow-up.

## Figures and Tables

**Table 1:** Perioperative Neurological Complications

Cerebrovascular Complications	5-11%
Ischemic stroke	
Hemorrhagic stroke	
Post-anoxic encephalopathy	
Transient Ischemic Attack	
Seizures	2 %
Peripheral neuropathy or myopathy	4 %
Brachial plexopathy	
Peroneal mononeuropathy	
Vocal cord paralysis	
Delirium or encephalopathy	2-9 %
Reversible posterior leukoencephalopathy	1 %
Progressive multifocal leukoencephalopathy	1 %
Herpes Zoster Radiculopathy	2 %
Cryptosporidium Meningitis	1 %

**Table 2:** Neurological Complications After the Perioperative Period

Cerebrovascular diseases	9%
Seizures	4%
Polyneuropathy	18%
Sleeping disorders	27%
Cognitive empairment	6%
Pain	30%
Depression	28%

**Table 3:** Type of Cerebrovascular Complications

Ischemic stroke	60%
TIA	28%
Hemorrhagic stroke	12%
Hypoxic encephalopathy	1%
Hyperperfusion syndrome	-
Vascular cognitive impairment	-

**Table 4:** Oxfordshire Classification

Total Anterior Cerebral Infarction	23,1%
Partial Anterior Cerebral Infarction	38,4%
Lacunar Cerebral Infarction	38,4%
Posterior Cerebral Infarction	23,1%

**Table 5:** TOAST Etiologic Classification

Large artery atherosclerosis	15,4%
Cardioembolism	14,4%
Small vessel disease	15,4%
Unusual causes	15,4%
Undetermined cause	38,4%

## References

[1] Almenar Bonet L (2009). Registro Español de Trasplante Cardiaco. XX Informe Oficial (1984-2008). Rev Esp Cardiol.

[2] van de Beek D, Kremers W, Daly RC (2008). Effect of Neurologic Complications on Outcome After Heart Transplant. Arch Neurol.

[3] Pérez-Miralles JC, Sánchez-Manso L, Almenar-Bonet T, Sevilla-Mantecón L, Martínez-Dolz Vílchez-Padilla JJ (2005). Incidence of and risk factors for neurologic complications after heart transplantation. Trasplant Proc.

[4] Jarquin-Valdivia AA, Wijdicks EFM, McGregor C (1999). Neurologic complications following heart transplantation in the modern era: decreased incidence, but postoperative stroke remains prevalent. Trasplant Proc.

[5] Belvís R, Martí-Fàbregas J, Cocho D (2005). Cerebrovascular disease as a complication of cardiac transplantation. Cerebrovasc Dis.

[6] Cemillán CA, Alonso-Pulpón L, Burgos-Lázaro R, Millán-Hernández I, del Ser T, Liaño-Martínez H (2004). Complicaciones neurológicas en una serie de 205 trasplantes cardíacos ortotópicos. Rev Neurol.

[7] Adair JC, Call GK, O’Connell JB, Baringer JR (1992). Cerebrovascular syndromes following cardiac transplantation. Neurology.

[8] Muraoka R, Yokota M, Aoshima M, Kyoku I, Nomoto S, Kobayashi A (1981). Subclinical changes in brain morphology following cardiac operations as reflected by computed tomographic scans of the brain. J Thorac Cardiovasc Surg.

[9] Rorick MB, Furlan AJ (1990). Risk of cardiac surgery in patients with prior stroke. Neurology.

[10] Tsukui H, Abla A, Teuteberg JJ (2007). Cerebrovascular accidents in patients with a ventricular assist device. J Thorac Cardiovasc Surg.

[11] Massaro AR, Dutra AP, Almeida DR, Diniz RV, Malheiros SM (2006). Transcranial Doppler assessment of cerebral blood flow: effect of cardiac transplantation. Neurology.

[12] van de Beek D, Kremers WK, Kushwaha SS, McGregor CG, Wijdicks EF (2009). No major neurologic complications with sirolimus use in heart transplant recipients. Mayo Clin Proc.

